# Mental health conditions in Mexico: Diagnosis trends based on hospital records

**DOI:** 10.1016/j.aprim.2026.103501

**Published:** 2026-04-23

**Authors:** Antonio Reyna-Sevilla, Rosibel Rodríguez-Bolaños, Oscar Alejandro Palacios-Rodríguez, Roxana Gámez-Ortiz, Miguel Galarde-López, Ilse Haide Ortega Ibarra

**Affiliations:** a Dirección de Prestaciones Médicas, Instituto Mexicano del Seguro Social, Ciudad de México, Mexico; b Departamento de Salud Reproductiva, Instituto Nacional de Salud Pública, Cuernavaca, Mexico; c Facultad de Psicología, Universidad Autónoma de San Luis Potosí, San Luis Potosí, Mexico; d Escuela de Salud Pública de México, Instituto Nacional de Salud Pública, Cuernavaca, Mexico; e Centro Nacional de Investigación Disciplinaria en Salud Animal e Inocuidad, Instituto Nacional de Investigaciones Forestales, Agrícolas and Pecuarias, Ciudad de México, Mexico; f Centro de Investigación en Alimentos y Nutrición, Universidad del Istmo, Oaxaca, Mexico

**Keywords:** Mental health conditions, Anxiety disorders, Depression, Mental disorders, Mexico, Condiciones de salud mental, Trastorno de ansiedad, Depresión, Trastornos mentales, México

## Abstract

**Objective:**

Analyze spatiotemporal trends in mental disorder diagnoses in Mexico between 2019 and 2023.

**Design:**

An ecological study was conducted based on records of mental disorder diagnoses in the population aged ≥21 years with social security coverage.

**Site:**

Mexico, based on data from the National Open Data Platform on primary care units of the Mexican Social Security Institute (IMSS).

**Participants:**

A total of 12,561,531 records obtained from diagnoses of mental disorders in the population aged ≥21 years with social security coverage during the period 2019–2023.

**Interventions:**

Rates were estimated, adjusted for sex and age. Spatio-temporal trends were evaluated using Moran's *I* index.

**Main measures:**

Classification of mental disorder, sex, age, entity, year.

**Results:**

At the national level, the mental disorder rate rose from 2419 per 100,000 population in 2019 to 7192 in 2023. Anxiety and obsessive–compulsive disorders, substance use disorders, and depressive episodes were the main diagnosis in both sexes. The data show a growing trend in mental disorders of various etiologies, in addition to depression and anxiety, which are treated by healthcare services. These disorders exhibited regional and gender-based variations during the study period.

**Conclusions:**

It is recommended to expand health service coverage to strengthen the detection and diagnosis of mental disorder, reinforce primary care in regions at greatest risk, and incorporate prevention strategies for disorders that may increase in magnitude (dementias, Alzheimer's disease, schizophrenia, and delusional disorders).

## Abstract (es)

**Objetivo:**

Analizar las tendencias espaciotemporales de los diagnósticos de trastornos mentales en México durante el período 2019-2023.

**Diseño:**

Se realizó un estudio ecológico basado en los registros de diagnósticos de trastornos mentales en la población con seguro social de ≥21 años.

**Emplazamiento:**

Mexico, a partir de datos de la Plataforma Nacional de Datos Abiertos sobre unidades médicas de primer nivel del Instituto Mexicano del Seguro Social (IMSS).

**Participantes:**

Un total de 12,561,531 registros obtenidos de los diagnósticos de trastornos mentales en la población ≥21 años con seguridad social durante el período 2019-2023.

**Intervenciones:**

Se estimaron las tasas, ajustadas por sexo y edad. Las tendencias espaciotemporales se evaluaron utilizando Moran I.

**Mediciones principales:**

Clasificación del desorden mental, sexo, edad, entidad, año.

**Resultados:**

A nivel nacional, la tasa de trastornos mentales aumentó de 2419 por cada 100 000 habitantes en 2019 a 7192 en 2023. Los trastornos de ansiedad y obsesivo-compulsivos; abuso de sustancias y episodios depresivos fueron los principales diagnósticos en ambos sexos. Los datos muestran una tendencia al alza en los trastornos mentales de diversas etiologías, además de la depresión y la ansiedad, que son tratados por los servicios sanitarios. Estos trastornos mostraron variaciones regionales y de género durante el periodo de estudio.

**Conclusiones:**

Se recomienda ampliar la cobertura de servicios de salud para fortalecer la detección y diagnóstico de trastornos mentales, reforzar la atención primaria en las regiones de mayor riesgo, además de incorporar estrategias de prevención para trastornos que podrían incrementar su magnitud (dementias; Alzheimer's disease; schizophrenia and delusional disorders).

## Introduction

Mental health is an essential and integral component of people's health. However, in most countries, health systems often lack sufficient resources to adequately address mental health needs,^1^ resulting in delayed diagnosis and limited access to care. Consequently, a substantial proportion of individual experience mental disorders without timely identification or treatment, despite mental health being recognized as a fundamental human right.^1^ Mental disorders are defined clinically significant disturbances in cognition, emotional regulation or behavior, reflecting dysfunctions in the psychological, biological or developmental processes underlying mental functioning.^2^, ^3^ These conditions are often associated with significant distress or impairment in various areas of daily life.^2^, ^4^ Generating descriptive epidemiological evidence on mental, behavioral and neurodevelopmental disorders in Mexico is therefore essential to inform evidence-based public policies and strengthen equitable access to care.

Globally, an estimated 970 million people worldwide were living with a mental disorder in 2019, with anxiety and depression being the most prevalent conditions.^1^, ^2^, ^5^, ^6^, ^7^ In the Region of the Americas, mental, neurological, substance use disorders, along with suicide, account for nearly one third of the years of life lost (YLL).^2^, ^5^ This burden contrasts with relatively low level of investment, as average expenditure on mental health represents only 2.8% of the total health budget, with marked inequalities between countries.^5^ In Latin America and the Caribbean, the growing impact of noncommunicable diseases has been documented in the context of the epidemiological and demographic transition, including accelerated population aging.^8^ Mental disorders often have an early onset and chronic course and contribute substantially to disability, particularly among adults in middle and older age groups.^9^, ^10^

In Mexico, mental disorders represent a significant and growing public health concern. Between 2019 and 2021, a 15.4% increase in DALYs attributable to mental disorders was reported, mainly driven by depressive and anxiety.^11^ Consistently, national report based on data from primary care units and hospitals have identified anxiety (51.5%) and depression (25.9%) as the most frequently diagnosed conditions.^12^ Despite existing evidence on the prevalence of mental disorders in Mexico.^11^, ^13^, ^14^, ^15^ Few studies have examined their national trends, particularly in the post-pandemic context.^1^, ^4^, ^6^, ^16^ In this context, the aim of this study was to analyze the spatiotemporal trends of mental disorders reported by a health institution in Mexico during the period 2019–2023.

## Methods

### Study design, population and data source

An ecological study was conducted based on the records of mental disorders of population with social insurance ≥21 years of age of the Mexican Institute of Social Security (IMSS). The data was obtained for all 32 states in Mexico between January 2019 and December 2023 from records concentrated in information systems of medical units that are part of the National Open Data Platform, which can be consulted at the following link: https://datos.gob.mx/dataset/deteccion_padecimientos_delegacion. Population data for each of the 32 states were analyzed in aggregate by entity. Therefore, based on the epidemiological design, it cannot be assumed that group trends apply to individuals. This requires caution when interpreting the results to avoid ecological fallacy.

### Screening and diagnosis of mental disorders

At IMSS, the process of diagnosing mental health conditions is carried out in stages at the primary care level. It consists of three phases: detection, clinical evaluation and final registration. The process begins with the timely detection of symptoms through a clinical interview and an assessment of overall functioning using the Global Assessment of Functioning (GAF) scale. A comprehensive clinical assessment is then carried out in accordance with the International Classification of Diseases (ICD-10), evaluating the severity of the condition, and particularly the risk of suicide. The diagnosis is then documented and the appropriate course of care defined: management and follow-up at the primary care level for mild and moderate cases, or immediate referral to emergency services and secondary or tertiary psychiatric care for severe dysfunction or psychiatric emergency.^17^, ^18^ Finally, the results are integrated into a nominal information system that collates the diagnoses recorded in each primary care unit, enabling analysis at state level.

The analysis is based on the frequency of diagnoses recorded by state, sex, and year. It is therefore not possible to identify individuals uniquely or control repeated diagnoses within the same person during the period studied (2019–2023).

### Statistical and geospatial analysis

Absolute and relative frequencies of mental disorders diagnoses were estimated according to sex, year and state. Data analysis was performed using Stata Statistical Software Release (STATA Corporation, Texas, United States) Stata 15.^19^ The trend of mental disorders diagnoses per 100,000 population ≥21 years of age during the study period was analyzed to evaluate annual variations from rates adjusted by sex, year and state. The analysis of mental disorders was heterogeneous because the ICD-10 classifies mental disorders with a hybrid clinical-dimensional approach. Choropleth maps were elaborated in QGIS (Creative Commons Corporation, California, United States) software version 3.22.3.^20^

The local Moran *I* statistic, also known as local spatial cluster analysis,^21^ was used to assess the magnitude, location, and spatial variations in the rates of mental disorders diagnosis. Due to the nature of the data (irregular polygons), a spatial weighting matrix based on the queen contiguity criterion was used to ensure at least one neighboring polygon (federal entity).

These rates, at state level, were classified into High–High or Low–Low categories, which indicated similar values in territorially close locations (positive autocorrelation), or High–Low and Low–High, which indicated states with different values in neighboring locations (negative autocorrelation). These categories made it possible to determine whether the spatial distribution of the high or low values followed a concentrated, dispersed or random pattern (*p* < 0.05) compared to the average rate for each year,^21^, ^22^ to identify whether the states with the highest values were located territorially close to each other and their behavior remained the same, or whether there were changes in the location of the patterns.

The results were presented in spatial cluster maps,^21^, ^22^ which made it possible to identify the states that integrated the spatial patterns, and which could suggest areas with a higher risk of mental disorders, based on the trends observed. Spatial cluster analysis was performed using Geoda software,^23^ while the final editing of the results using maps was performed using QGIS software (Creative Commons Corporation, California, United States) software version 3.22.3.^20^

## Results

Between 2019 and 2023, a total of 12,561,531 diagnoses of mental disorders were registered among individuals aged ≥21 years with IMSS social insurance in Mexico (median: 2,286,328; interquartile range: 1,763,185–3,306,919). At the national level, the diagnosis rate rose from 2419 per 100,000 population in 2019 to 7192 in 2023 (Table 1). All states registered higher rates in 2023 than in 2019, with differences in magnitude. The largest relative increases were observed in Tabasco and Tamaulipas (4.4-fold), followed by Zacatecas (4.0-fold) and Chiapas (3.6-fold). In 2023, the highest rates were recorded in Aguascalientes (11,854 per 100,000), Nayarit (10,597), Michoacán (10,591), and Mexico City (9304).

**Table 1:** Rate ratios of mental disorder diagnoses per 100,000 population ≥21 years of age with IMSS social insurance, Mexico; 2019–2023.

State	2019	2020	2021	2022	2023	Relation 2019–2023
Tabasco	1550	2352	3191	4510	6822	4.4
Tamaulipas	1307	1890	2478	3464	5732	4.4
Zacatecas	1918	2689	3485	4855	7700	4.0
Chiapas	1346	2044	2815	4452	4855	3.6
Estado de México	1703	2539	3338	4531	5989	3.5
Quintana Roo	1368	2202	2837	4006	4463	3.3
Coahuila	1518	2266	3033	4547	4915	3.2
Hidalgo	1550	2203	2955	4094	4916	3.2
Nayarit	3268	4689	5950	8257	10,597	3.2
Veracruz	2520	3747	4953	7006	8146	3.2
Aguascalientes	3820	5388	6715	9182	11,854	3.1
CDMX	3041	4347	5587	7948	9304	3.1
Baja California Sur	2294	3530	4547	6560	6983	3.0
Guerrero	3185	4874	6318	8825	9495	3.0
Oaxaca	1451	2019	2765	4041	4342	3.0
Querétaro	1822	2685	3448	4837	5504	3.0
Tlaxcala	1931	2848	3834	5493	5828	3.0
Yucatán	3039	4161	5288	6922	8986	3.0
**National**	**2419**	**3481**	**4469**	**6203**	**7192**	**2.9**
Durango	2992	4237	5496	7737	8802	2.9
Colima	2617	3621	4576	6995	7470	2.9
Guanajuato	2267	3249	4127	5713	6519	2.9
Morelos	2441	3491	4643	6649	7143	2.9
Puebla	2221	3282	4317	5945	6356	2.9
San Luis Potosí	2225	3166	4018	5645	6123	2.8
Campeche	2728	3903	5136	7230	7352	2.7
Chihuahua	3104	4410	5654	7947	8413	2.7
Jalisco	3327	4720	5801	7669	8977	2.7
Sonora	2404	3357	4304	6322	6571	2.7
Baja California	2801	4005	5108	6878	7229	2.6
Michoacán	3997	5499	6957	9194	10,591	2.6
Nuevo León	2062	2863	3630	5049	5344	2.6
Sinaloa	2754	3904	4868	6805	7160	2.6
Mean (Min.–Max.)	2392 (1307–3997)	3443 (1890–5499)	4442 (2477–6957)	6228 (3463–9194)	7202 (4341–11,853)	

**Figure 1:**
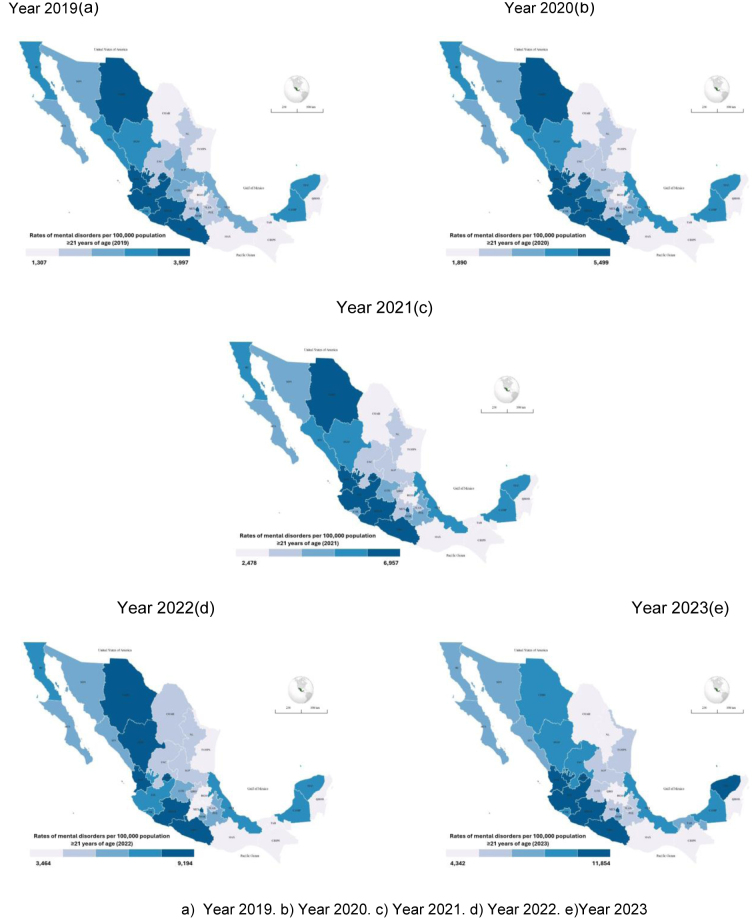
Spatiotemporal trends in rates of mental disorders per 100,000 population ≥21 years of age with IMSS social insurance, Mexico; 2019–2023.

Fig. 1 shows diagnosis rates by state, ranging from 3997 to 11,854 per 100,000 population aged ≥21 years with social insurance. States in the northern, western, and southern regions were classified in the highest quintile throughout the study period. In 2023, Yucatán was also classified in this quintile.

**Figure 2:**
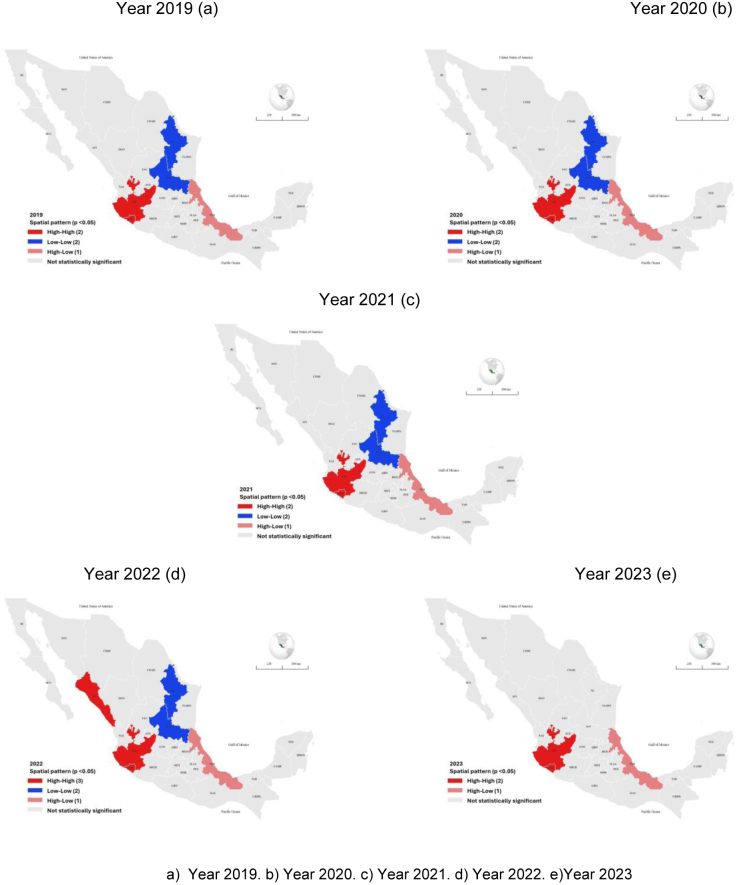
Spatial clusters of the rate of diagnoses of mental disorders per 100,000 population ≥21 years of age with IMSS social insurance, Mexico; 2019–2023.

Fig. 2 presents the spatial cluster analysis. Jalisco and Colima in the western region, and Veracruz in the eastern region, were classified in the High–High or High–Low categories (*p* < 0.05). Nuevo León and San Luis Potosí were classified in the Low–Low category (*p* < 0.05).

**Figure 3:**
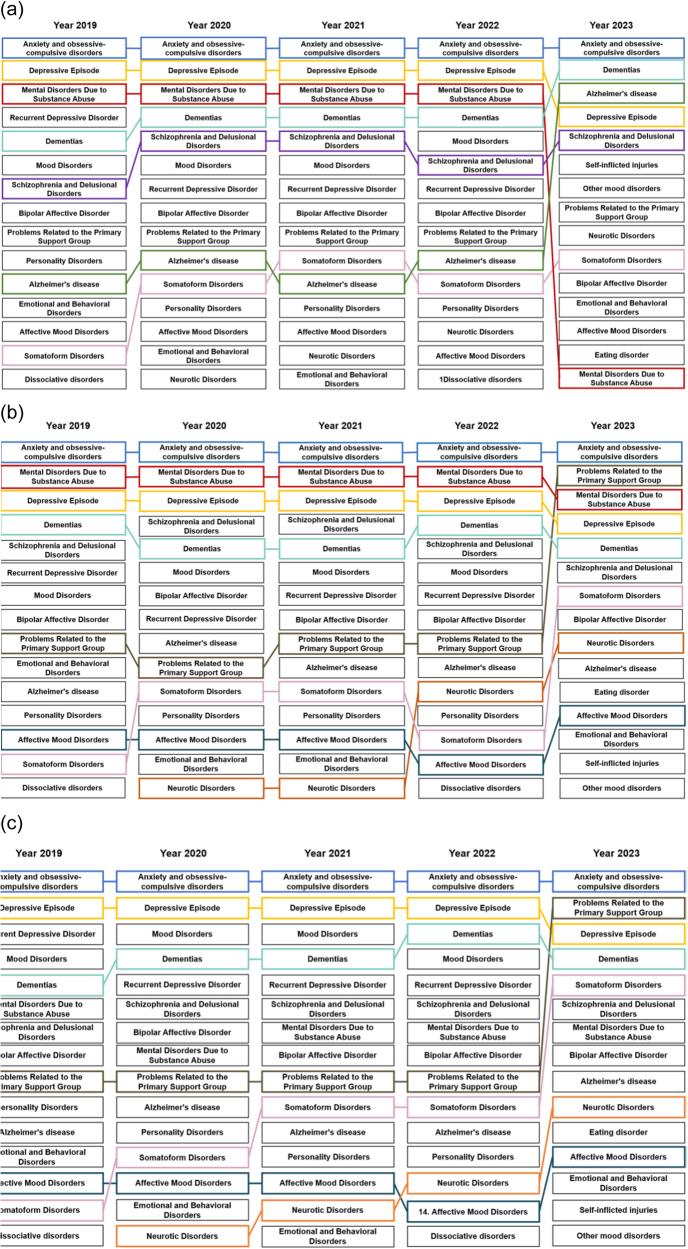
Ranking of the main diagnoses of mental disorders reported in population ≥21 years with social security attended at the first level of care of the IMSS, 2019–2023. (a) Ranking of the main diagnoses of mental disorders reported in population ≥21 years. (b) Ranking of the main diagnoses of mental disorders reported in women ≥21 years. (c) Ranking of the main diagnoses of mental disorders reported in men ≥21 years.

Fig. 3 show the distribution of mental health disorder diagnoses by sex. In both women and men (Fig. 3b and c), anxiety and obsessive–compulsive disorders ranked as the most frequent diagnoses. Among women, depressive episodes were also among the most frequent conditions. In 2023, problems related to the primary support group ranked second and somatoform disorders ranked fifth. Among men, substance use-related mental disorders ranked second overall and third in 2023, while problems related to the primary support group ranked second in that year.

**Figure 4:**
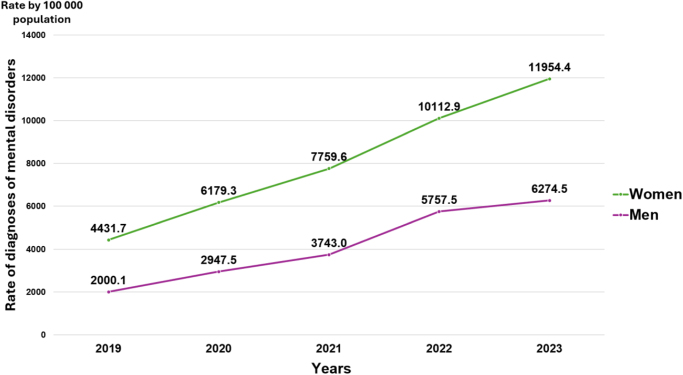
Rate of diagnoses of mental disorders per 100,000 population ≥21 years with social security attended at the first level of care of the IMSS, by sex in Mexico; 2019–2023.

Fig. 4 shows diagnosis rates by sex. Among women, rates ranged from 4432 per 100,000 in 2019 to 11,954 in 2023. Among men, rates ranged from approximately 2000 per 100,000 in 2019 to 6274 in 2023.

## Discussion

The results document an increase in the number and rate of mental health disorder diagnoses among adults receiving care at the IMSS between 2019 and 2023. This increase was observed in all states, although with differences in magnitude and spatial distribution, including the presence of geographic clusters. The diagnoses recorded were primarily related to anxiety and obsessive–compulsive disorders in both women and men, while depressive episodes were more frequent among women and substance use disorders among men. These findings describe an increasing volume and diversity of mental health conditions managed within healthcare services, as well as variations by region and sex during the study period.

The observed increase in mental health disorder diagnoses coincides temporally with the COVID-19 pandemic period. Although the present analysis does not allow causal inferences, previous studies have reported increases in diagnoses or service utilization related to depression, anxiety, suicidal ideation, and substance use during this period.^1^, ^6^, ^11^, ^16^, ^24^, ^25^ These changes have been discussed in relation to social restrictions, disruptions in daily activities, and modifications in healthcare utilization patterns. In this context, periodic updates of administrative health records remain relevant for monitoring trends in mental health service utilization and for informing the planning and organization of mental health services within the health system.^7^, ^16^, ^25^

Marked geographic differences in diagnosis rates were identified across states, with heterogeneity in both magnitude and spatial distribution. Some states with higher rates or larger relative increases coincide with regions characterized by unfavorable sociodemographic conditions, including income inequality, illiteracy, or indicators of social violence, as documented in previous studies.^26^, ^27^, ^28^, ^29^, ^30^ However, these factors were not directly analyzed in the present study and should therefore be interpreted as contextual characteristics rather than explanatory variables.^16^, ^31^, ^32^ Further analytical studies incorporating sociodemographic indicators are needed to explore these associations in the Mexican context.

Dementia-related diagnoses, including Alzheimer's disease, were among the mental disorders identified during the study period. Their presence in the diagnostic distribution could be interpreted within the context of population aging in Mexico, which has been documented in national studies conducted prior to and during the pandemic period.^33^ Previous research has reported increasing mortality and prevalence Alzheimer's disease among older adults.^6^, ^34^, ^35^ As age-specific analyses were not included in the present study, these observations should be considered exploratory.

Sex differences in diagnostic patterns were also observed. Anxiety and obsessive–compulsive disorders were the most frequent diagnoses in both women and men, while depressive episodes were more frequent among women and substance use-related disorders among men. These findings are consistent with previous studies reporting sex differences in the distribution of this mental disorders.^6^, ^11^, ^13^, ^36^, ^37^, ^38^ Nevertheless, the absence of age-specific and other sociodemographic variables in the present analysis limits more detailed comparisons with existing evidence.

Overall, the results are consistent with reports from other countries, although direct comparisons should be interpreted with caution due to differences in population structure, data sources, health system organization, and the timing and impact of the COVID-19 pandemic.^39^, ^40^ The findings describe the frecuence of mental disorders of diverse etiology within primary care services and underscore the relevance of first-level care as a key point of contact for the identification, diagnosis, and initial management of mental health conditions across the adult population. In low- and middle-income countries, previous reports have documented substantial gaps in access to mental health care, particularly at early stages of the disease course.^11^, ^15^, ^25^, ^38^, ^41^

Several limitations should be considered. First, the analysis was based on secondary administrative data from a single institution within the Mexican National Health System, which limits the generalizability of the results to other health subsystems and to the population. The use of routine health information systems inherently reflects patterns of service provision and utilization rather than true population prevalence, introducing potential access-to-care bias, as individuals with limited or no access to mental health services are likely underrepresented.^42^, ^43^ Furthermore, some diagnoses may not have been established by mental health specialists, and variability in clinical practices, diagnostic criteria, and coding procedures across regions and levels of care may have contributed to diagnostic heterogeneity, potentially resulting in underestimation or misclassification of mental disorders.^36^

Another important limitation is the absence of key sociodemographic and contextual variables, such as age group, occupation, or specific etiological factors, which restricted a more detailed characterization of mental health needs and limited the possibility of subgroup analyses. Additionally, the observed increase in diagnosis rates during the post-pandemic period may partially reflect improved detection, increased mental health awareness, and changes in help-seeking behavior, rather than a true increase in incidence, as has been documented in other settings following the COVID-19 pandemic.^44^ Despite these constraints, similar temporal trends and patterns have been reported in other national studies using different data sources, supporting the external consistency and robustness of the findings.^12^ Despite these limitations, this study provides a recent epidemiological overview of mental health diagnoses recorded in primary care units across all 32 states of Mexico over a five-year period.^30^

## Conclusion

Between 2019 and 2023, rates of mental disorder diagnoses recorded in IMSS primary care units increased across all states of Mexico, with variability by sex and geographic region. Anxiety and obsessive–compulsive disorders were the most frequently diagnosed conditions in both women and men, while depressive episodes were more common among women and substance use-related disorders among men. These results describe the growing demand for mental health care and highlight the central role of identification and initial management of mental conditions.

**What is known about this topic**

• Globally, mental disorders represent the leading cause of years lived with disability (DALYs).
• The COVID-19 pandemic increased mental health disorders.
• Anxiety and depression account for the greatest burden of DALYs among the Mexican population aged 15–49.

**Contributions of this study**

• A growing number of mental health disorders beyond anxiety and depression are being diagnosed.
• Mental health diagnoses and care must be accessible to people of all ages.
• The increase in mental health patterns may be determined by some geographic territories.

## Authorship contribution statement

ARS, RGO, and MGL collected, tabulated and analyzed the data, ARS, RRB, OAPR, RGO, MGL, and IHOI participated in writing. ARS, RGO and MGL analyzed the data, coordinated and guided the production of the study. ARS, RRB, OAPR, RGO, MGL, IHOI participated in the writing, formatting and submission process. All authors read and approved of the final manuscript.

## Ethics approval and consent to participate

As the study involved analyzing secondary epidemiological data and the research centers’ protocols regarding the publication of patient data were followed, ensuring the privacy of the participants, approval from the local Ethics Committee was not required. However, the study complied with current Mexican health research regulations. The datasets used and analyzed during the current study are available in https://datos.gob.mx/dataset/deteccion_padecimientos_delegacion.

## Use of AI

Not applicable.

## Funding

Not funding.

## Conflict of interest

The authors declare that they have no competing interests.

## References

[1] WHO (2022). https://www.who.int/publications/i/item/9789240050860.

[2] World Health Organization (2020). https://icd.who.int/browse11/l-m/en.%20Available%20from:%20https://icd.who.int/browse/2024-01/mms/en#334423054.

[3] World Health Organization (2024). https://iris.who.int/bitstream/handle/10665/375767/9789240077263-eng.pdf?sequence=1.

[4] Gordillo A., Andre T., Medici C., Calvo N. (2023). Mental health among adolescents and young adults in latin america and the caribbean. World Bank Publ.

[5] Institute for Health Metrics and Evaluation (2024). https://vizhub.healthdata.org/gbd-compare/.

[6] Pan American Health Organization (2021).

[7] Rahim F., Dzhusupov K., Qasim N.H., Zhumagaliuly A., Bodnar N., Khozhamkul R. (2023). https://www.researchsquare.com/article/rs-3257421/v1.

[8] Kohn R., Levav I., de Almeida J.M.C., Vicente B., Andrade L., Caraveo-Anduaga J.J. (2005). Los trastornos mentales en América Latina y el Caribe: asunto prioritario para la salud pública. Rev Panam Salud Pública.

[9] Borges G., María, Medina-Mora E., En D., Soc P., López-Moreno S. (2004 Sep 8). The role of epidemiologyin mental disorder research. Salud Publica Mex.

[10] Rodríguez J.J., Kohn R., Aguilar Gaxiola S. (2010). Epidemiología de los trastornos mentales en América Latina y el Caribe. Rev Fac Nac Salud Pública.

[11] Medina-Mora M.E., Orozco R., Rafful C., Cordero M., Bishai J., Ferrari A. (2023). Los trastornos mentales en México 1990–2021. Resultados del estudio Global Burden of Disease 2021. Gac Med Mex.

[12] CONASAMA (2024).

[13] Shamah-Levy T., Romero-Martínez M., Barrientos-Gutiérrez T., Cuevas-Nasu L., Bautista-Arredondo S., Colchero M. (2022). https://ensanut.insp.mx/.

[14] García-Pacheco J.Á., Torres Ortega M.d.L., Borges G. (2024). The burden of mental disorders in Mexico, 1990-2019: Mental and neurological disorders, substance use, suicides, and related somatic disorders. Spanish J Psychiatry Ment Heal.

[15] Vázquez-Salas A., Hubert C., Portillo-Romero A., Valdez-Santiago R., Barrientos-Gutiérrez T., Villalobos A. (2023). Sintomatología depresiva en adolescentes y adultos mexicanos: Ensanut 2022. Salud Publica Mex.

[16] Kwong A.S.F., Pearson R.M., Adams M.J., Northstone K., Tilling K., Smith D. (2021). Mental health before and during the COVID-19 pandemic in two longitudinal UK population cohorts. Br J Psychiatry.

[17] IMSS. Programa IMSS-Bienestar 1979–2025. Servicio de Atención Integral a la Salud Mental (SAISME). 2025. p. 1. Available from: https://www.imss.gob.mx/imss-bienestar/saisme [cited 17.2.26].

[18] IMSS. Diagnóstico y manejo de los principales trastornos mentales en medicina familiar y psicología. 2025. p. 25. Available from: https://educacionensalud.imss.gob.mx/ces_wp/wp-content/uploads/2021/08/Brochure_Mini_Guia_Diagnostico_Trastornos_Mentales_VF_19072021.pdf [cited 17.2.26].

[19] StataCorp. Stata Statistical Software: Release 14. College Station, TX: StataCorp LP. 2015. 2015. Available from: https://www.stata.com/support/faqs/resources/citing-software-documentation-faqs/ [cited 21.5.20].

[20] Develop Team QGIS. QGIS. 2021. https://qgis.org/. Available from: https://qgis.org/.

[21] Fritz C.E., Schuurman N., Robertson C., Lear S. (2013). A scoping review of spatial cluster analysis techniques for point-event data. Geospat Health.

[22] Auchincloss A.H., Gebreab S.Y., Mair C., Diez Roux A.V. (2012). A review of spatial methods in epidemiology, 2000–2010. Annu Rev Public Health.

[23] Anselin L. (2005). Exploring spatial data with geoda: a workbook center for spatially integrated social science. Cent Spat Integr Soc Sci.

[24] Antiporta D.A., Bruni A. (2020). Emerging mental health challenges, strategies, and opportunities in the context of the COVID-19 pandemic: Perspectives from South American decision-makers. Rev Panam Salud Pública.

[25] Genis-Mendoza A.D., Martínez-Magaña J.J., López-Narváez M.L., González-Castro T.B., Juárez-Rojop I.E., Nicolini H. (2021). Mental health problems due to social isolation during the COVID-19 pandemic in a Mexican population. Front Public Heal.

[26] INEGI. Vol. 2020, Censo de Población y Vivienda 2020. 2020. p. 22-4 Censo de Población y Vivienda 2020. Available from: https://www.inegi.org.mx/contenidos/programas/ccpv/2020/doc/resumen_ejecutivo_2020.pdf [cited 29.5.20].

[27] Stockdale S.E., Wells K.B., Tang L., Belin T.R., Zhang L., Sherbourne C.D. (2007). The importance of social context: Neighborhood stressors, stress-buffering mechanisms, and alcohol, drug, and mental health disorders. Soc Sci Med.

[28] Casas Patiño D., Rodríguez Torres A., Salazar Morales M.R. (2016). Violence in Mexico: a social or public health problem?. Medwave.

[29] Puyana J.C., Puyana J.C.J., Rubiano A.M., Montenegro J.H., Estebanez G.O., Sanchez A.I. (2017). https://pubmed.ncbi.nlm.nih.gov/28329741/.

[30] González Block M., Reyes Morales H., Hurtado L.C., Balandrán A., Méndez E. (2020). Mexico: health system review. Health Syst Transit.

[31] Kessler R.C., Aguilar-Gaxiola S., Alonso J., Benjet C., Bromet E.J., Cardoso G. (2017). Trauma and PTSD in the WHO world mental health surveys. Eur J Psychotraumatol.

[32] Benjet C., Sampson L., Yu S., Kessler R.C., Zaslavsky A., Evans-Lacko S. (2019). Associations between neighborhood-level violence and individual mental disorders: Results from the World Mental Health surveys in five Latin American cities. Psychiatry Res.

[33] Celis-De la Rosa A.d.J., Cabrera-Pivaral C.E., Báez-Báez M.G.L., Celis-Orozco A., Gabriel-Ortiz G., Zavala-González M.A. (2018). Mortalidad por enfermedad de Alzheimer en México de 1980 a 2014. Gac Med Mex.

[34] (2024). Instituto Nacional de Geriatría. Plan Nacional de Demencia México 2024.

[35] World Health Organization (WHO) (2025). https://www.who.int/news-room/fact-sheets/detail/dementia.

[36] Agudelo-Botero M., Giraldo-Rodríguez L., Rojas-Russell M., González-Robledo M.C., Balderas-Miranda J.T., Castillo-Rangel D. (2021). Prevalence, incidence and years of life adjusted for disability due to depressive disorders in Mexico: Results of the Global Burden of Disease Study 2019. J Affect Disord Rep.

[37] Medina-Mora M.E., Borges G., Benjet C., Lara C., Berglund P. (2007). Psychiatric disorders in Mexico: Lifetime prevalence in a nationally representative sample. Br J Psychiatry.

[38] WHO (2021). Comprehensive MH action plan 2013–2020. News.

[39] Karnecki K., Gos T., Steiner J., Mańkowski D., Kaliszan M. (2023). Epidemiology of suicide in the Tri-City metropolitan area in Poland in 2010–2019. Eur Arch Psychiatry Clin Neurosci.

[40] Kohn R., Ali A.A., Puac-Polanco V., Figueroa C., López-Soto V., Morgan K. (2018). Mental health in the Americas: an overview of the treatment gap. Rev Panam Salud Publica/Pan Am J Public Heal.

[41] Barrón-Velázquez E., Mendoza-Velásquez J.J., Mercado-Lara A., Quijada-Gaytán J.M., Flores-Vázquez J.F. (2024). The Mental Health Provider Shortage in the Mexican Public Sector: 2023 estimates of psychiatrists and psychologists. Salud Ment.

[42] Patel V., Prince M. (2010). Global mental health: a new global health field comes of age. JAMA.

[43] Health Organization World (2021). https://www.who.int/publications/i/item/9789240036703.

[44] Moreno C., Wykes T., Galderisi S., Nordentoft M., Crossley N., Jones N. (2020). How mental health care should change as a consequence of the COVID-19 pandemic. Lancet Psychiatry.

